# Quercitrin Ameliorates Hyperlipidemia and Hepatic Steatosis in Ovariectomized Mice

**DOI:** 10.3390/life10100243

**Published:** 2020-10-15

**Authors:** Haeng Jeon Hur, Yeon-Hui Jeong, Sang Hee Lee, Mi Jeong Sung

**Affiliations:** Research Group of Natural Materials and Metabolism, Food Functionality Research, Korea Food Research Institute, Jeollabuk-Do 55365, Korea; mistltoe@kfri.re.kr (H.J.H.); Yeon-hui@kfri.re.kr (Y.-H.J.); shlee@kfri.re.kr (S.H.L.)

**Keywords:** quercitrin, lipid metabolism, estrogen deficiency, hepatic steatosis, inflammation, nonalcoholic fatty liver disease

## Abstract

Nonalcoholic fatty liver disease (NAFLD) is associated with progressive metabolic diseases. Estrogen deficiency increases the NAFLD risk among postmenopausal women. Thus, effective agents to prevent and treat NAFLD in postmenopausal women are required. Quercitrin (Quer) is a natural glycosylated flavonoid with antimicrobial, anti-inflammatory, and hypolipidemic effects. This study investigated whether Quer improves dysregulated lipid metabolism and suppresses hepatic steatosis in ovariectomized (OVX) mice as an experimental model mimicking postmenopausal women. Mice were assigned to the following four groups: SHAM, OVX, OVX + β-estradiol (0.4 mg/kg diet), and OVX + Quer (500 mg/kg diet). Mice were administered a diet with or without Quer for three months. OVX mice displayed significantly higher body mass, epidermal fat, and liver weights than those of SHAM mice. However, these levels were reduced in Quer-treated mice. Quer treatment reduced the levels of serum lipid metabolites, including triglycerides, total cholesterol, and low-density lipoprotein cholesterol. Furthermore, Quer reduced liver lipid steatosis and inhibited the expression of proinflammatory cytokines, such as tumor necrosis factor-α, IL-6, and IL-1β. The results of the present study indicate that Quer improves dysregulated lipid metabolism and reduces hepatic steatosis and inflammation by compensating for estrogen deficiency, suggesting that Quer may potentially exert protective effects during hepatic steatosis in postmenopausal women.

## 1. Introduction

Nonalcoholic fatty liver disease (NAFLD), one of the most common liver diseases, is characterized by excessive liver lipid deposition without alcohol consumption [1,2]. NAFLD severity changes from simple steatosis to nonalcoholic steatohepatitis (NASH), fibrosis, and cirrhosis. NAFLD is mediated by metabolic diseases, including obesity, insulin resistance, and dyslipidemia [3]. Estrogen (ES) is directly associated with liver lipid metabolism, and its decline markedly increases body weight gain, insulin resistance, hyperlipidemia, and hepatic steatosis, all of which are associated with NAFLD [4]. Therefore, postmenopausal women present accelerated progression of hyperlipidemia and NAFLD [5]. Hence, estrogen deficiency is a potentially important risk factor underlying the development of NAFLD in postmenopausal women.

Phytoestrogens are naturally occurring compounds in plants that resemble human estrogens in their chemical structure and pharmacological activity [6,7]. Phytoestrogens include isoflavones, lignans, flavonoids, and coumestans [8]. Flavonoids, commonly found in apples, grapes, onions, and herbs, exhibit potential beneficial effects on human health, including anti-allergic, antiviral, antioxidant, and anti-inflammatory activities, and they have been shown to have preventive and therapeutic effects against NAFLD [9]. Moreover, dietary flavonoid consumption improves dyslipidemia and decreases hepatic fat accumulation [10,11].

Quercitrin (3,5,7,3′,4′-OH, 3-rhamnosylquercetin, Quer), a glycosylated form of quercetin, is a naturally available type of flavonoid used in dietary supplements and as a dietary ingredient. Quer has several pharmacological activities, including antimicrobial, anti-inflammatory, and hypolipidemic effects [12,13,14]. However, numerous studies have focused on the aglycone (quercetin) form, and limited information is available regarding the biological effects of glycoside forms, such as the potential of flavonoids in the treatment of NAFLD [15]. This study aimed to investigate the beneficial effect of Quer on hyperlipidemia and hepatic steatosis induced by estrogen deficiency in an experimental model of dysregulated lipid metabolism in postmenopausal women.

## 2. Materials and Methods

### 2.1. Animal Studies

All animal experiments were approved by the KFRI Animal Care and Use Committee (KFRI-IACUC, KFRI-M-16064). Female C57BL/6 mice (8-week-old) were purchased from Charles River, Korea (Seoul, Korea). All animals were housed under standardized conditions (22 °C ± 2 °C) with a 12/12 h light/dark cycle and were given access to tap water and food. Quer (purity > 98%) was purchased from Chengdu Biopurify Phytochemicals Ltd. (Chengdu, China), and β-estradiol (dissolved in corn oil) was purchased from Sigma Chemical Corp. (St. Louis, MO, USA). The mice were subjected to sham surgery or were ovariectomized. After 1 week, the mice were divided into the following four groups: (1) sham with vehicle treatment (SHAM, N = 10); (2) ovariectomized (OVX) mice treated with vehicle (OVX, N = 10); (3) OVX mice treated with β-estradiol (OVX + ES, N = 10, 0.4 mg/kg diet); and (4) OVX mice treated with Quer (OVX + Quer, N = 10, 500 mg/kg in diet). The diets were combined with the AIN-93G diet (Dyets Inc., Bethlehem, PA, USA), which is formulated to support growth in mice (Table 1). The diets were administered for 3 months. Body weight and food intake were assessed weekly. Blood was sampled through abdominal aortal puncture, and epidermal fat and liver tissue were dissected and weighed.

### 2.2. Serum Analysis

Serum total cholesterol (TC), triglycerides (TG), and high-density lipoprotein cholesterol (HDL-C) concentrations were estimated enzymatically by commercial kits (Asan Pharmaceuticals, Hwasung, Korea), in accordance with the manufacturer’s instructions. LDL cholesterol (LDL-C) concentration was determined as follows: LDL-C = TC − HDL-C − TG/5.

### 2.3. Body Fat Composition and Liver Histological Analysis

Body and fat mass were determined through dual energy X-ray absorptiometry (DXA) (InAlyzer dual X-ray absorptiometry, Medikors, Seongnam, Korea). Liver tissue samples were dissected, fixed in 10% formalin, and embedded in paraffin. Five micrometer thick liver blocks were cut and stained with hematoxylin and eosin (H&E). The NAFLD activity score (NAS) was quantified as the sum of individual scores for steatosis (0–3) and lobular inflammation (0–2) [16]. The sections were examined under a Nikon Eclipse 80i microscope (Nikon Imaging, Inc., Seoul, Korea). A total of five images from each slide were analyzed.

### 2.4. Real-Time PCR

Total RNA was purified from the liver tissue using the RNeasy mini Kit (Qiagen, Valencia, CA, USA), in accordance with the supplier’s protocols. RNA was reverse transcribed into cDNA using the ReverTra Ace qPCR RT Master Mix (ToYoBo, Osaka, Japan). Quantitative real-time PCR analysis was analyzed using an AB ViiA 7 real-time PCR system (Applied Biosystems, Foster City, CA, USA). The primer pairs were as follows: IL-1β sense, 5′-TGT AAT GAA AGA CGG CAC ACC-3′; IL-1β antisense, 5′-TCT TCT TTG GGT ATT GCT TGG-3′; IL-6 sense, 5′-TGG AGT ACC ATA GCT ACC TGG A-3′; IL-6 antisense, 5′-TGA CTC CAG CTT ATC TGT TAG GAG-3′; TNF-α sense, 5′-ACC CTC ACA CTC AGA TCA TC-3′; TNF-α antisense, 5′-GAG TAG ACA AGG TAC AAC CC-3′; glyceraldehyde 3-phosphate dehydrogenase (GAPDH) sense, 5′-AAA TGG TGA AGC TCG CTC TG-3′; and GAPDH antisense, 5′-TGA AGG GGT CGT TGA TGG-3′. The mRNA expression levels were normalized to GAPDH expression.

### 2.5. Statistical Analysis

Statistical analyses were performed using Prism 8.0 (GraphPad Software, San Diego, CA, USA). All data are expressed as mean ± SEM values. Mean comparisons between two groups were performed for significant differences using ANOVA, followed by Tukey’s post hoc test; *p*-values < 0.05 were considered significant. 

## 3. Results

### 3.1. Quercitrin Inhibited Body Weight Gain, Epidermal Fat, and Liver Weight in OVX Mice

The initial body weight was not significantly different among the groups. After 12 weeks, body weight gains markedly decreased in OVX mice as compared with SHAM mice (*p* < 0.001) and were significantly lower in Quer-treated mice than in OVX mice (*p* < 0.01). Furthermore, epidermal fat and liver weight were markedly higher in OVX mice as compared with SHAM mice (*p* < 0.001). However, Quer-treated mice had significantly lower epidermal fat and liver weight than those of OVX mice (*p* < 0.05) (Table 2). Furthermore, as expected, OVX + ES mice showed lower body weight gain (*p* < 0.05), epidermal fat (*p* < 0.001), and liver weight (*p* < 0.01) than those of OVX mice.

### 3.2. Quercitrin Disrupted the Distribution of Fat Mass and Body Mass in OVX Mice

**Figure 1:**
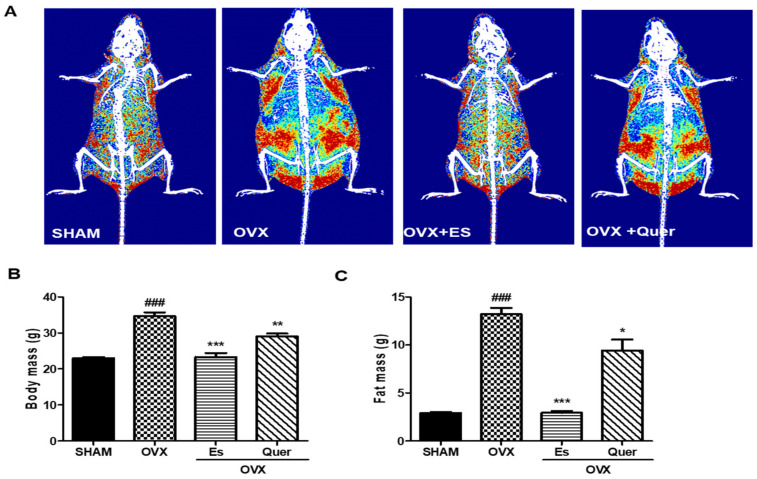
Effect of quercitrin on the distribution of fat mass and body mass in OVX mice. (**A**) The radiograph of body fat is represented by dual energy X-ray absorptiometry (DXA) images of mice. Red indicates high-density fat, yellow indicates intermediate-density fat, and blue indicates low-density fat; (**B**) Body mass; (**C**) Fat mass. All values are expressed as mean ± SEM values. ^###^ *p* < 0.001 vs. control group. * *p* < 0.05, ** *p* < 0.01, and *** *p* < 0.001 vs. OVX group (N = 10 per group).

To investigate the effects of Quer on body composition, body mass, and fat mass, DXA was performed. Images of all groups are displayed as radiographs of body fat distribution. OVX mice displayed larger red areas rather than blue and yellow areas, indicating a higher fat density than that of areas with low and intermediate fat densities. Quer-treated mice displayed a lower fat density than that of OVX mice. The body (*p* < 0.01) and fat mass (*p* < 0.05) distribution in Quer-treated mice was significantly lower than those in OVX mice (Figure 1).

### 3.3. Quercitrin Suppressed Serum Lipid Metabolite Levels in OVX Mice

To investigate the effect of Quer on serum lipid metabolite levels, we assessed serum TG, TC, HDL-C, and LDL-C levels. TC (*p* < 0.001), LDL-C (*p* < 0.001), and TG (*p* < 0.05) levels were significantly higher in OVX mice than in SHAM mice. However, Quer-treated mice displayed markedly lower serum TC (*p* < 0.01), LDL-C (*p* < 0.01), and TG (*p* < 0.05) levels. Furthermore, serum HDL-C levels were not significantly different among any group. These results indicate that Quer regulated dyslipidemia in OVX-induced mice (Table 3).

### 3.4. Quercitrin Decreased Liver Injury in OVX Mice

**Figure 2:**
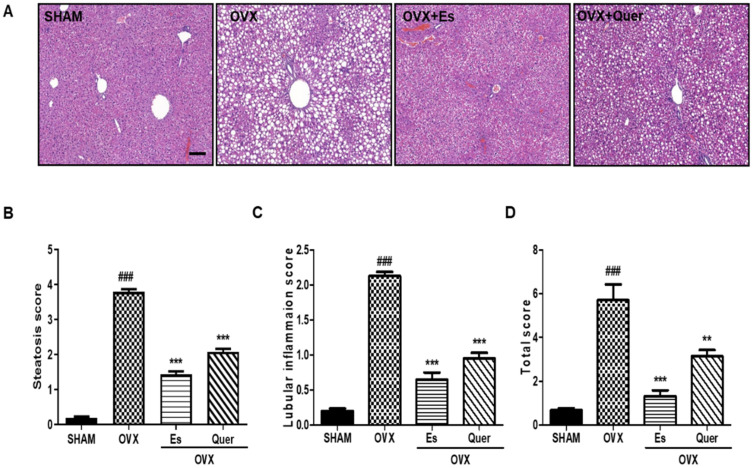
Effect of quercitrin on lipid accumulation in the liver and hepatic steatosis in OVX mice. (**A**) Liver tissues were fixed and stained with hematoxylin-eosin (H&E). Images were examined by light microscopy (**B**) Steatosis score; (**C**) Lobular inflammation score; and (**D**) Total score, were determined. Scale bar = 200 μm. All values are expressed as mean ± SEM. ^###^ *p* < 0.001 vs. control group. ** *p* < 0.01 and *** *p* < 0.001 vs. OVX group (N = 10 per group).

To investigate the effect of Quer on OVX-induced fat accumulation in the liver, we performed H&E staining and determined the histopathological score. OVX mice displayed markedly greater micro-/macrovascular steatosis and lobular inflammation than SHAM mice did (*p* < 0.001). However, Quer-treated mice had significantly less micro-/macrovascular steatosis and lobular inflammation than OVX mice had (*p* < 0.001). These results indicate that Quer suppressed liver steatosis in OVX mice (Figure 2).

### 3.5. Quercitrin Inhibited the Expression of Hepatic Inflammatory Molecules in OVX Mice

**Figure 3:**
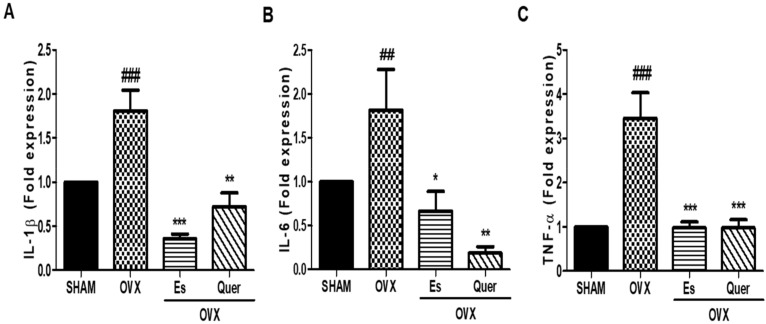
Effect of quercitrin on liver inflammatory cytokines in OVX mice. Total mRNA levels in liver tissues from each group were quantified through real-time PCR. Relative expression levels of (**A**) IL-1β; (**B**) IL-6; and (**C**) TNF-α, were determined and normalized to those of glyceraldehyde 3-phosphate dehydrogenase (GAPDH). All values are expressed as mean ± SEM. ^##^ *p* < 0.01 and ^###^ *p* < 0.001 vs. control group. * *p* < 0.05, ** *p* < 0.01, and *** *p* < 0.001 vs. OVX group (N = 10 per group).

To evaluate the effect of Quer on the expression of hepatic inflammatory molecules, we quantified the expression levels of proinflammatory cytokines, including IL-1β, IL-6, and TNF-α, via real-time PCR analysis. Consequently, IL-1β (*p* < 0.01), IL-6 (*p* < 0.01), and TNF-α (*p* < 0.001) expression levels were markedly lower in Quer-treated mice than in OVX mice. ES treatment further downregulated these genes in OVX mice (Figure 3).

## 4. Discussion

Estrogen regulates energy and lipid metabolism, and thus estrogen deficiency potentially disrupts energy metabolism and lipid accumulation in women [17,18]. NAFLD is the most common chronic liver disease, the progression of which is accelerated in postmenopausal women, accompanied by markedly increased lipid metabolism and the development of obesity and related metabolic diseases, including dyslipidemia, obesity, and insulin resistance [19]. Therefore, estrogen deficiency is a prominent risk factor in the pathophysiology of NAFLD.

Studies targeting postmenopausal women have involved ovariectomized animal models as experimental models [20]. In OVX rodents, obesity is increased, and other metabolic diseases are induced, including dyslipidemia and NAFLD. OVX mice commonly demonstrate increased body weight owing to excessive fat accumulation, as well as increased liver lipid accumulation, leading to fatty liver [21,22]. Therefore, OVX mice show increased body fat and liver weight. In the present study, OVX mice had dramatically increased body fat, and liver weights and developed hepatic steatosis. However, Quer treatment inhibited these increases. These results suggest that Quer might ameliorate obesity and NAFLD in OVX mice.

Menopausal women and OVX rodents present one or more risk factors mediated by NAFLD development, such as obesity, insulin resistance, and dyslipidemia [21,23]. In the development of NAFLD, lipid accumulation in the liver can be traced by increased free fatty acid uptake and de novo lipogenesis for increased TG synthesis, as well as impaired fatty acid beta oxidation [24]. In particular, elevated serum levels of TG lead to intrahepatic lipid accumulation [25,26], as well as increased LDL-C and decreased HDL-C levels in OVX animals [21]. In the present study, the OVX group displayed elevated TC, LDL-C, and TG levels and reduced HDL-C levels. However, Quer supplementation markedly reversed these changes (Table 3). Furthermore, these results suggest that Quer inhibits OVX-induced hyperlipidemia. However, a limitation of this study is that we have only shown changes in serum lipids (TG, HDL-C, and LDL-C) mediated by hepatic accumulation; in further studies, we intend to investigate the correlation between lipogenesis and lipid metabolism on the basis of their expression patterns.

Accumulating evidence indicates that postmenopausal women and OVX animal models are more susceptible to NAFLD risk. During NAFLD pathogenesis, histopathological changes occur in the liver [21,27,28]. Nanashima et al. reported that OVX mice presented fat accumulation, ballooning degeneration, and infiltration of inflammatory cells [21]. Furthermore, infiltration of inflammatory cells in the liver predominantly synthesized proinflammatory cytokines, including TNF-α, IL-1β, and IL-6 [29]. Several studies have reported that the suppression of TNF-α, IL-1β, and IL-6 can inhibit intrahepatic lipid accumulation [30,31]. The present study findings indicate that Quer significantly inhibits intrahepatic fat accumulation; inflammatory cell infiltration; and TNF-α, IL-1β, and IL-6 expression (Figure 2 and Figure 3). These data suggest that Quer supplementation protects against liver steatosis and hepatic inflammation by inhibiting proinflammatory cytokines, thus suppressing NAFLD associated with hepatic lipid accumulation and liver injury in OVX mice.

Quercetin is the most common flavonoid in nature, and it is mainly present in glycosylated forms such as Quer [12]. Quer has several biological activities, such as anti-inflammatory, antioxidant, and hypolipidemic effects [12,13,14]. In order to explain the dosage used at the beginning, daily flavonoid intake (typically present in onion, apple, grape, wine, herbs and spices) in human diet is highly variable, with estimations ranging from 23 mg/day [32] to more than 500 mg/day [33]. Animal studies have reported that Quer concentrations of 1–100 mg/kg body weight have pharmacological effects [34,35,36]. Among these dosages, we selected 50 mg/kg body weight. However, we fed Quer through the diet instead of oral gavage. Therefore, we calculated the diet dose according to the diet dose calculator (https://www.researchdiets.com) [37]. For diet dose calculation, we input the following parameters: single daily dose, 40 mg/bw/day; body weight, 20 g; and daily food intake, 2 g. Finally, the diet dose showed 500 mg/kg diet, which we used for this study. The Quer concentration of 50 mg/kg body weight is approximately similar to 500 mg/kg in the diet.

The results of the present study indicate that Quer is an effective alternative agent for preventing lipid metabolism dysregulation and hepatic steatosis in OVX mice as an experimental model for postmenopausal women. These results suggest that Quer could be potentially used as a supplement for postmenopausal women with NAFLD.

## Figures and Tables

**Table 1:** Formulation of the AIN-93G diet.

Ingredient	SH, OVX	OVX + ES	OVX + Qtr
Cornstarch	397.486	397.486	397.486
Casein	200.000	200.000	200.000
Dextrin	132.000	132.000	132.000
Sucrose	100.000	100.000	100.000
Soybean oil (no additives)	70.000	70.000	70.000
Fiber	50.000	50.000	50.000
Mineral mix (AIN-93G-MX)	35.000	35.000	35.000
Vitamin mix (AIN-93-vx)	10.000	10.000	10.000
l-Cystine	3.000	3.000	3.000
Choline bitartrate	2.500	2.500	2.500
*tert*-Butylhydroquinone	0.014	0.014	0.014
Quer	0	0	0.5
Es	0	0.0004	0
Total	1000	1000.0004	1000.5

**Table 2:** Effect of quercitrin on body weight gain, epidermal fat, and liver weight in ovariectomized (OVX) mice. Body weight gain, epidermal fat mass, and liver mass were determined after 11 weeks of quercitrin (500 mg/kg of diet) and estradiol (0.4 mg/kg of diet) treatment in female OVX mice. All values are expressed as mean ± SEM.

Parameters	SHAM	OVX	OVX + ES	OVX + Quer
Initial body weight (g)	19.53 ± 0.76	19.81 ± 1.15	19.63 ± 0.85	19.57 ± 1.06
Weight gain (g)	3.13 ± 0.5	14.10 ± 1.03 ^###^	4.49 ± 1.01 *	8.59 ± 1.23 **
Epidermal fat (g)	0.258 ± 0.027	1.17 ± 0.162 ^###^	0.172 ± 0.025 ***	0.942 ± 0.163 *
Liver (g)	0.13 ± 0.13	1.89 ± 0.33 ^###^	1.25 ± 0.13 **	1.33 ± 0.12 *

^###^*p* < 0.001 vs. control group. * *p* < 0.05, ** *p* < 0.01, and *** *p* < 0.001 vs. OVX group (N = 10 per group).

**Table 3:** Effect of quercitrin on serum lipid parameters in dyslipidemia in OVX mice. Total cholesterol (TC), high-density lipoprotein cholesterol (HDL-C), low-density lipoprotein cholesterol (LDL-C), and triglycerides (TG). All values are expressed as mean ± SEM.

Parameters	SHAM	OVX	OVX + ES	OVX + Quer
TC (mg/dL)	128.0 ± 9.8	215.3 ± 13.9 **^###^**	175.3 ± 10.7 **	171.0 ± 9.9 **
HDL-C (mg/dL)	106.7 ± 11.3	86.7 ± 5.2	87.6 ± 7.2	104.3 ± 12
LDL-C (mg/dL)	28.9 ± 2.3	108.6 ± 12.5 **^###^**	93.3 ± 9.4	79.7 ± 15.4 **
TG (mg/dL)	62.4 ± 6.3	80.9 ± 6.5 **^#^**	100.7 ± 10.6	66.1 ± 6.4 *

^#^*p* < 0.05, ^###^ *p* < 0.001 vs. control group. * *p* < 0.05 and ** *p* < 0.01 vs. OVX group (N = 10 per group).
